# Relationship of fibroblast growth factor 21, Klotho, and diabetic retinopathy: a meta-analysis

**DOI:** 10.3389/fendo.2024.1390035

**Published:** 2024-08-27

**Authors:** Yanhua Jiang, Weilai Zhang, Yao Xu, Xiandong Zeng, Xin Sun

**Affiliations:** ^1^ Department of Ophthalmology, Fourth People’s Hospital of Shenyang, Shenyang, China; ^2^ Department of Ophthalmology, Fourth Affiliated Hospital of Soochow University, Suzhou, China; ^3^ Department of Endocrinology, First Affiliated Hospital of Soochow University, Suzhou, China

**Keywords:** FGF21, Klotho, diabetic retinopathy, fibroblast growth factor 21, DR

## Abstract

**Background:**

Diabetic retinopathy (DR) is a serious microvascular complication of diabetes mellitus. Research has identified a close relationship between fibroblast growth factor 21 (FGF21) and DR. FGF21 is a member of the FGF subfamily, which is activated by the Klotho coenzyme involved in the occurrence of DR. However, the association between FGF21, Klotho, and DR remains controversial.

**Aim:**

To assess FGF21 and Klotho levels in patients with DR.

**Methods:**

A literature search of the Web of Science, Wiley Online Library, PubMed, China National Knowledge Infrastructure and Wanfang databases was performed. The title or abstract search terms “diabetic retinopathy” and “DR” were used in combination with “fibroblast growth factor 21”, “FGF21”, and “Klotho”. Meta-analysis results are presented as standardized mean difference (SMD) with corresponding 95% confidence interval (CI).

**Results:**

Fifteen studies were included in this meta-analysis. FGF21 levels in patients with DR were significantly higher than in non-DR patients with diabetes (SMD: 2.12, 95% CI [1.40, 2.84]). Klotho levels in patients with DR were significantly lower than in non-DR patients with diabetes (SMD: –0.63, 95% CI [–1.22, – 0.04]).

**Conclusions:**

This systematic review is the first to evaluate the relationship between FGF21, Klotho levels, and DR. FGF21 levels were significantly higher in patients with DR. Fully elucidating the role of FGF21 will significantly contribute to the treatment of DR.

## Introduction

Diabetes is a common metabolic disease characterized by abnormally high blood glucose levels. Epidemiological studies have reported that diabetes not only has an increasing annual incidence rate, but is also projected to become the seventh leading cause of death worldwide by 2030 (1), posing a serious threat to human health. Diabetic retinopathy (DR) is a serious microvascular complication of diabetes. In 2020, approximately 103 million individuals were affected by DR globally, and this number is projected to increase to 160 million by 2045 (2). DR is an ischemic disease characterized by early stage retinal neurodegeneration, retinal microaneurysms, and bleeding, as well as possible accompanying “cotton wool” patches, venous bead-like changes, and retinal microvascular abnormalities (3). DR is the main cause of visual dysfunction and blindness in working-age adults worldwide and is significantly associated with a risk for future cerebrovascular accidents, myocardial infarction, and congestive heart failure (4). Conventional treatment methods for DR, such as retinal laser photocoagulation, hypoglycemic drugs, and anti-vascular endothelial growth factor (anti-VEGF) therapy, are ineffective and are accompanied by numerous side effects (5). It is clear that DR imposes a significant public health burden worldwide, seriously threatening the vision and quality of life of affected individuals. As such, there is an urgent need to further clarify the precise pathological and physiological mechanisms of DR to improve prevention strategies for DR and develop new treatment strategies.

Recent research has identified a close relationship between fibroblast growth factor 21 (FGF21) and DR, which may be a therapeutic target for pathological vascular growth in patients (6–8). FGF21 is a member of the FGF subfamily, with a coding gene located on chromosome 19 that encodes a long-chain protein comprising 209 amino acids. FGF21 binds to FGF receptors 1–4 under the action of the Klotho coenzyme and enters the bloodstream, producing effects by binding to different receptors (9, 10). FGF21 is expressed in multiple tissues and organs of the human body, mainly in the liver, followed by the pancreas, adipose tissue, myocardial cells, skeletal muscles (11). FGF21, which participates in metabolic regulation in the bloodstream, originates mainly from the liver. However, the association between FGF21, Klotho, and DR remains controversial. The FGF21 and Klotho levels of DR patients were various among studies. To our knowledge, this systematic review is the first to evaluate the relationship between FGF21 and Klotho levels and DR.

## Methods

### Literature search

A literature search of the Web of Science, Wiley Online Library, PubMed, China National Knowledge Infrastructure (CNKI) and Wanfang databases was performed. The title or abstract search terms “diabetic retinopathy” and “DR” were used in combination with “fibroblast growth factor 21”, “FGF21”, and “Klotho”. The focus of the search period was 1980 to 2024, with publication languages limited to English and Chinese. The reference lists of the retrieved articles were manually searched to identify additional, potentially eligible studies. To date, no studies have been conducted on this topic. This systematic review and meta-analysis was registered with PROSPERO under accession number CRD42024501425. All items that should be reported for systematic reviews and meta-analyses are listed in the **Supplementary Table 1**.

### Inclusion criteria

Meta-analysis was conducted on studies fulfilling the following criteria: sufficient data regarding FGF21, Klotho levels in DR patients and non-DR patients with diabetes; case-control design; and language limited to English and Chinese.

### Data extraction and risk of bias

As part of the preliminary screening process, 2 reviewers independently used the search strategy and read titles and abstracts to exclude studies that did not fulfill the inclusion criteria. To determine whether the studies met the inclusion criteria, the two reviewers methodologically reviewed the full text. If the author’s information is incomplete, they can contact and crosscheck the author. If the conclusions of the two evaluators were inconsistent, the differences were resolved through discussion. If the discussion failed to resolve any differences, it was judged and arbitrated by a third researcher. The Cochrane Collaboration recommends the Newcastle-Ottawa Scale (NOS) as a tool to assess bias in observational studies (12, 13). Studies were rated according to the NOS, which ranged from zero to nine stars, and star scores were used to determine quality. There are three aspects in the NOS: the method for selecting case and control groups, their comparability, and the method for assessing exposure.

### Statistical analysis

Heterogeneity among the included studies was assessed using the I^2^ statistic, and the data are expressed as standardized mean difference (SMD) and corresponding 95% confidence interval (CI). Fixed-effects models were used if I^2^ was < 50% and heterogeneity among studies was low or moderate; otherwise, random-effects models were used if I^2^ was > 50%. A sensitivity analysis was performed to evaluate the stability of the results. Begg’s and Egger’s tests were used to detect publication bias. *P* < 0.05 was set as the significance level. Data analysis was performed using Review Manager version 5.3. And the sensitivity analysis and publication bias were performed using Stata version 12.0.

## Results

### Literature search and study selection

**Figure 1:**
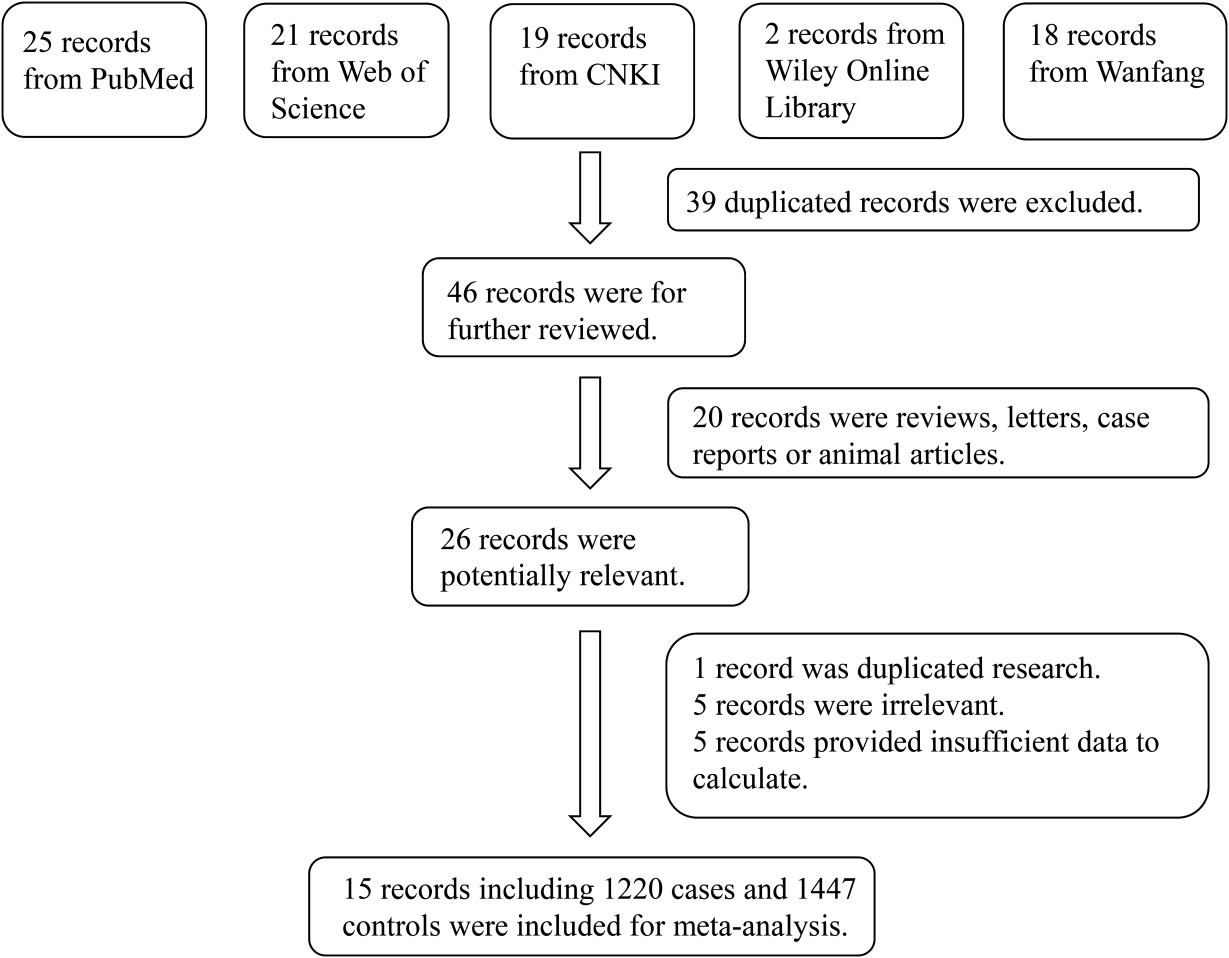
Flowchart of the detailed procedure for the inclusion or exclusion of selected studies.

The initial literature search retrieved 85 studies from the Web of Science, Wiley Online Library, PubMed, CNKI and databases. After screening, 15 studies including 1220 DR cases and 1447 controls were included (6, 7, 14–26). A flow-diagram illustrating the study selection process is presented in **Figure 1**. The characteristics of each of the included studies are summarized in **Table 1**. All 15 studies included in this meta-analysis fulfilled the criteria for the Newcastle-Ottawa Scale categories of selection, comparability, and exposure.

**Table 1:** Study characteristics of the published studies included in the meta-analysis.

Author	Publication Year	Study Period	Region	Number		Sex(M/F)		Age(years)		Sample	NOS
				Case	Control	Case	Control	Case	Control		
Lin Y	2014	October 2009 to May 2012	China	83	34	43/40	20/14	60.10 ± 11.70	59.40 ± 10.20	FGF21	7
Esteghamati A	2016		Iran	46	44	17/29	21/23	56.50 ± 9.00	55.00 ± 12.00	FGF21	5
Mousavi Z	2017	2016 to 2017	Iran	25	22	6/19	5/17	56.00 ± 7.00	54.0 ± 6.00	FGF21	6
Zhang L	2018	January 2016 to January 2018	China	99	127	48/51	59/68	52.6 ± 10.25	52.9 ± 8.25	Klotho	6
Li LJ	2019	March 2016 to March 2018	China	34	160	19/15	96/64	61. 35 ± 11. 22	58.48 ± 13.11	FGF21	6
Xu LZ	2020	January 2016 to December 2018	China	120	120	67/53	64/56	52.14 ± 12.61	51.44 ± 11.57	FGF21	6
Ji B	2020	November 2015 to November 2016	China	33	27	11/22	12/15	57.64 ± 6.23	58.00 ± 4.70	Klotho	6
Corcillo A	2020		UK	46	35			61.20 ± 8.80	60.70 ± 9.30	Klotho	5
Jin S	2021	January 2018 to October 2019	China	309	345	162/147	185/160	58.58 ± 10.31	57.11 ± 11.99	FGF21	7
Heidari Z	2021		Iran	91	93	34/57	32/61	55.47 ± 9.99	54.12 ± 11.27	FGF21	6
Cai LY	2021	February 2018 to April 2018	China	90	85	51/39	45/40	48.36 ± 6.14	48.41 ± 6.05	FGF21	6
Wan F	2022	January 2017 to December 2019	China	106	74	57/49	40/34	56.82 ± 10.13	56.34 ± 9.20	Klotho	5
Hu W	2022	November 2020 to October 2021	China	45	112	25/20	63/49	55.84 ± 10.44	52.95 ± 12.01	Klotho	6
Xiao T	2023	September 2020 to September 2022	China	63	101	33/30	52/49	53.94 ± 7.49	54.10 ± 8.36	FGF21	6
Gao YQ	2023	October 2019 to October 2022	China	30	68	16/14	38/30	53 .43 ± 10.37	54.01 ± 10. 56	FGF21	6

### Results of meta-analysis

**Figure 2:**
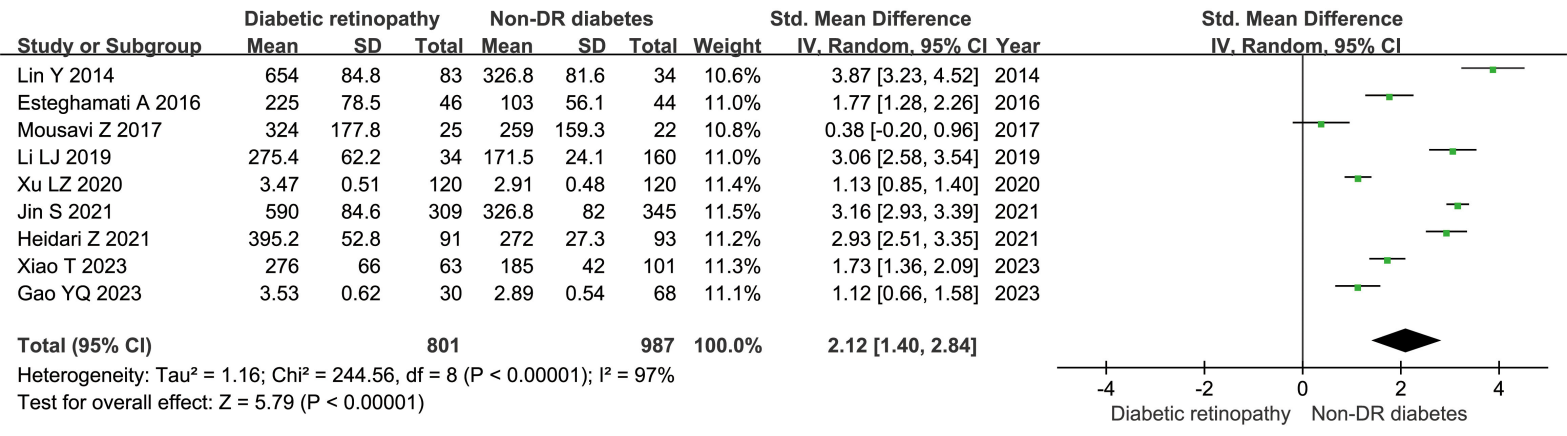
Forest plots of FGF21 level in patients with diabetic retinopathy compared to non-diabetic retinopathy diabetes patients. Diamond represents the pooled SMDs at 95% CI. SMD, standardized mean difference; CI, confidence interval.

**Figure 3:**
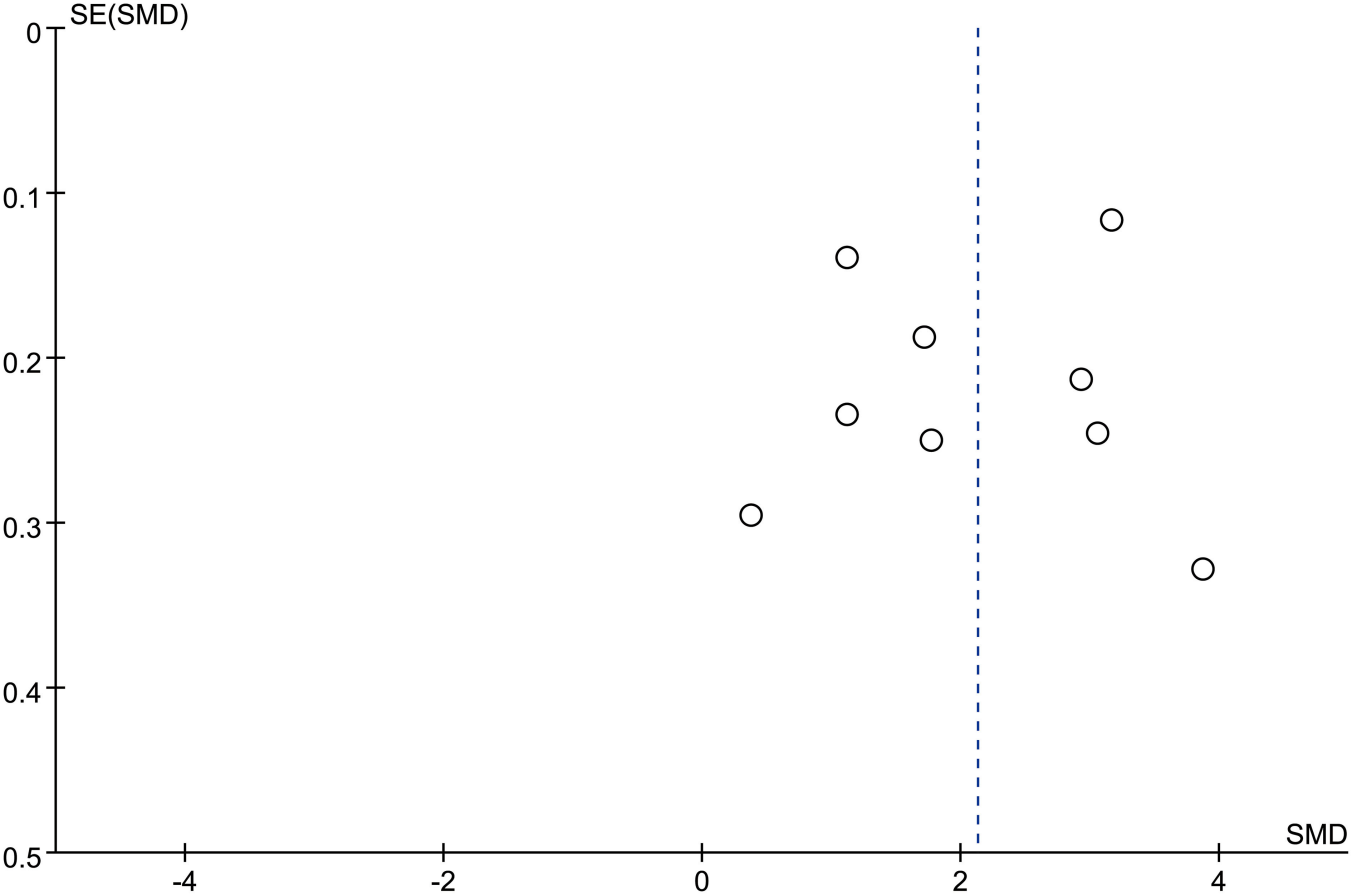
Funnel plots of FGF21 level in patients with diabetic retinopathy compared to non-diabetic retinopathy diabetes patients. SMD, standardized mean difference.

**Figure 4:**
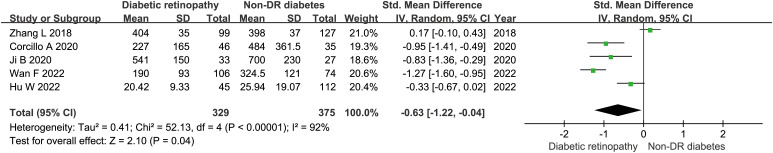
Forest plots of Klotho level in patients with diabetic retinopathy compared to non-diabetic retinopathy diabetes patients. Diamond represents the pooled SMDs at 95% CI. SMD, standardized mean difference; CI, confidence interval.

**Figure 5:**
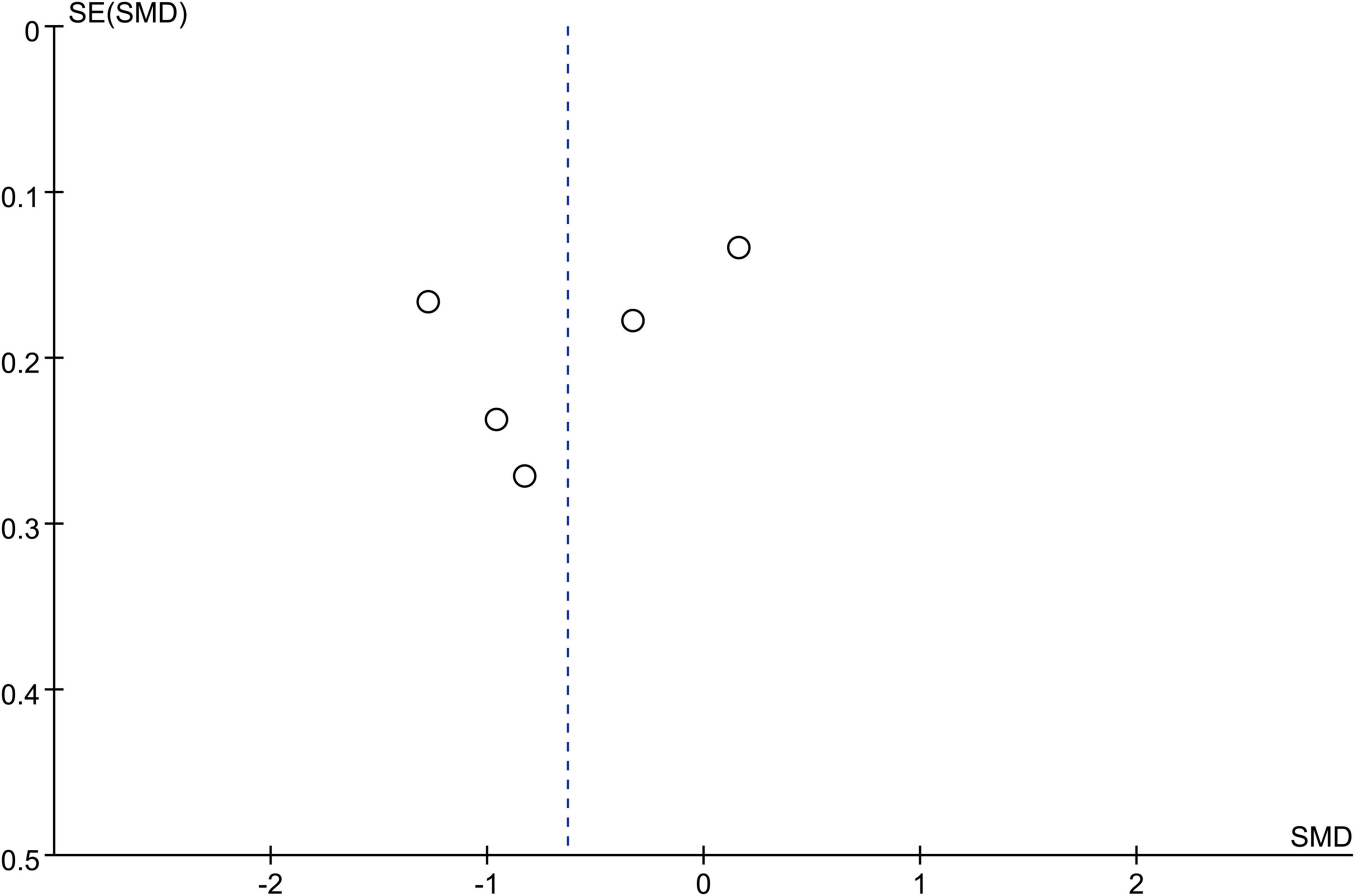
Funnel plots of Klotho level in patients with diabetic retinopathy compared to non-diabetic retinopathy diabetes patients. SMD, standardized mean difference.

There 10 articles were about FGF21 and DR. While, in these 10 articles, one article used healthy people as control group, not the patients with non-DR diabetes. So, we have performed the analysis in different control groups (non-DR diabetes patients and healthy controls). FGF21 levels were significantly higher in patients with DR than in non-DR patients with diabetes (SMD:2.12, 95% CI [1.40, 2.84]; I^2^ = 97%) (6, 7, 14, 16, 17, 20, 21, 24, 25). Forest plot and funnel plot of FGF21 levels in patients with DR compared to those in non-DR diabetes are presented in **Figures 2**, **3**. It was also found that FGF21 levels in patients with DR were significantly higher than that in healthy controls [SMD: 3.90, 95% CI (2.46, 5.35); I^2^ = 98%] (6, 7, 14, 17, 21, 22, 25). In addition, there was no difference in FGF21 levels between patients with non-proliferative DR (NPDR) and those with proliferative DR (PDR) [SMD: 0.89, 95% CI (–1.21, 2.99); I^2^ = 97%] (6, 14, 20–22). The Klotho level in patients with DR was significantly lower than that in non-DR patients with diabetes [SMD: –0.63, 95% CI (–1.22, – 0.04); I^2^ = 92%] (15, 18, 19, 23, 26). Forest plots and funnel plot of Klotho levels are presented in **Figures 4**, **5**.

### Sensitivity analysis and publication bias

Each study was subjected to a sensitivity analysis to determine its influence. Sensitivity analysis showed no significant differences from our previous estimates, indicating that a single study had a marginal impact on the overall estimate (**Supplementary Figures 1**, **2**). Accordingly, the meta-analysis yields stable results. A thorough and comprehensive search of the databases was conducted. Begg’s and Egger’s tests were conducted to identify whether publication bias was present in the reviewed studies. The results (*P* > 0.05) indicated that there was no publication bias (**Supplementary Figures 3**–**6**).

## Discussion

This systematic review is the first to evaluate FGF21 and Klotho levels in patients with DR. In our previous research, we found the leptin and chemerin levels in patients with DR were significantly higher than those in non-DR patients (27). In this meta-analysis, fourteen independent studies were included in the meta-analysis. We conclude that FGF21 levels were significantly higher in patients with DR than non-DR patients with diabetes, and that Klotho levels were significantly lower in patients with DR than non-DR patients with diabetes.

FGF21 is a key regulator of retinal lipid and glucose metabolism. It can metabolize lipoproteins in the adipose tissue and maintain adipocyte phospholipid homeostasis. FGF21 also increases lipid utilization during amino acid starvation. FGF21 acts by regulating the activities of peroxisome proliferator-activated receptors (PPARs) and peroxisome proliferator-activated receptor γ coactivator-1 (PGC-1). FGF21 is crucial in PPAR-α agonists to improve metabolic processes in obese mice (28). FGF21 inhibits the growth of pathological retinal neovascularization through adiponectin (APN). FGF21 promotes APN expression in the blood circulation in a dose-dependent manner (29). Fu et al. reported that APN could inhibit neovascularization of the retina and choroid in mice (30). The use of long-acting FGF21 analogs can increase the concentration of retinal APN in mice, indicating that FGF21 has important effects on metabolic functions (31). To determine whether APN mediates the protective effect of FGF21 on retinal neovascularization, the retinal vascular systems of mice with and without long-acting FGF21 analogs were compared under oxygen-induced retinopathy in *APN* gene knockout mice. Studies have shown that APN deficiency exacerbates retinal neovascularization and eliminates the beneficial effects of long-acting FGF21 analogs in reducing hypoxic retinal neovascularization. In addition, APN can inhibit retinal neovascularization by reducing the level of tumor necrosis-alpha (TNF-α) (32). In summary, FGF21 inhibits the growth of pathological retinal neovascularization by targeting APN and reducing TNF-α, which is a key risk factor for hypoxia-induced retinopathy (33).

High blood glucose levels can induce oxidative stress, which is a key factor in DR (34). Photosensitive cells are the most metabolically active cells in the human body and are prone to metabolic disorders and oxidative stress (35). Fu et al. (36) reported that the use of FGF21 in insulin-deficient diabetic mice reversed retinal neuron defects caused by diabetes, improved the function and morphology of photoreceptors, and reduced inflammation of photoreceptors. NRF2 regulates oxidative stress and inflammatory response, and interleukin (IL)-1β is an inflammatory factor causing retinal neurovascular injury. FGF21 protects photoreceptor cells from oxidative stress by upregulating the expression of the NRF2 protein in the DR retina and decreasing the production of IL-1β (37, 38). Photosensitive cells can release inflammatory products that stimulate the surrounding cells (39) and cause changes in retinal vascular permeability in diabetic mouse models (40). In addition, the effect of long-term FGF21 administration on the inhibition of retinal vascular leakage in *in vivo* and *in vitro* models has been verified (41). Retinal tissue is highly sensitive to metabolism, and photoreceptors contain the largest number of mitochondria in human cells. Among all cells in the retina, photoreceptors mount the largest response to retinal oxidative stress and inflammation caused by diabetes. In patients with PDR and subsequent retinitis pigmentosa, retinal neovascularization is slow. Therefore, enhancing the levels of antioxidants in photoreceptor cells can prevent neurovascular damage in DR; however, this is, to some extent, independent of the APN-TNF-α pathway (36).

Experimental evidence suggests that FGF21 is beneficial in DR. In the present study, we confirmed that FGF21 levels were significantly higher in patients with DR than in those without DR. It is difficult to explain this phenomenon in patients with DR. Some researchers have named this phenomenon “FGF21 resistance” (42, 43). Although this hypothesis is supported, the mechanism underlying FGF21 resistance has not yet been elucidated. Once the role of FGF21 is fully elucidated, however, we believe that it will contribute significantly to the treatment of DR.

The FGF21 coenzyme Klotho has been found to be expressed in the human retina, optic nerve, and lens (44, 45). Some evidence suggests that Klotho regulates many mechanisms involved in maintaining retinal cell homeostasis and function (44, 46, 47). First, Klotho-knockout mice exhibit several morphological changes compared with wild-type mice, including reduced pigmentation of the retinal pigment epithelium layer, enlarged choroidal vessels, thinning and deformation of the basement membrane, and signs of degeneration of the outer photoreceptor layer (46). Proteomic analysis has shown that proteins involved in eye development, visual perception, and mitochondrial function are downregulated in Klotho-knockout mice (47). Thus, Klotho-knockout mice have reduced retinal function, with functional defects comparable to those observed in insulin-like growth factor-1 knockout mice (44). Several experimental models have shown that depletion of Klotho negatively affects important functions of retinal cells, including oxidative stress, VEGF-A secretion, and phagocytosis, thereby activating mechanisms that may contribute to the onset and progression of DR. However, there is some evidence that treatment with recombinant Klotho improves retinal function.

The present study had some limitations. First, the duration of diabetes and disease severity varied among the included studies, and data regarding NPDR and PDR were limited. Second, the languages of literature search were limited to English and Chinese. As such, high-quality research investigating the role(s) of FGF21 and Klotho in the treatment of DR is warranted. It is, therefore, important to interpret the results of this meta-analysis cautiously.

## Conclusion

This systematic review is the first to evaluate the relationship between FGF21 and Klotho levels and DR. FGF21 levels were significantly higher in patients with DR. Fully characterizing the role of FGF21 will significantly contribute to the treatment of DR.

## Data Availability

The original contributions presented in the study are included in the article/**Supplementary Material**. Further inquiries can be directed to the corresponding authors.

## Supplementary material

Supplementary Table 1 Preferred reporting items for systematic review and meta-analyses (PRISMA) checklist.

Supplementary Figure 1 The sensitivity analysis results of FGF21 level in patients with diabetic retinopathy compared to non-diabetic retinopathy diabetes patients.

Supplementary Figure 2 The sensitivity analysis results of Klotho level in patients with diabetic retinopathy compared to non-diabetic retinopathy diabetes patients.

Supplementary Figure 3 The Begg’s test of FGF21 level in patients with diabetic retinopathy compared to non-diabetic retinopathy diabetes patients.

Supplementary Figure 4 The Egger’s test of FGF21 level in patients with diabetic retinopathy compared to non-diabetic retinopathy diabetes patients.

Supplementary Figure 5 The Begg’s test of Klotho level in patients with diabetic retinopathy compared to non-diabetic retinopathy diabetes patients.

Supplementary Figure 6 The Egger’s test of Klotho level in patients with diabetic retinopathy compared to non-diabetic retinopathy diabetes patients.
