# Supplementary figures and images for: Relationship of fibroblast growth factor 21, Klotho, and diabetic retinopathy: a meta-analysis
Source: Front Endocrinol (Lausanne). 2024 Aug 27;15:1390035. doi: 10.3389/fendo.2024.1390035 (PMC11384578; doi:10.3389/fendo.2024.1390035)

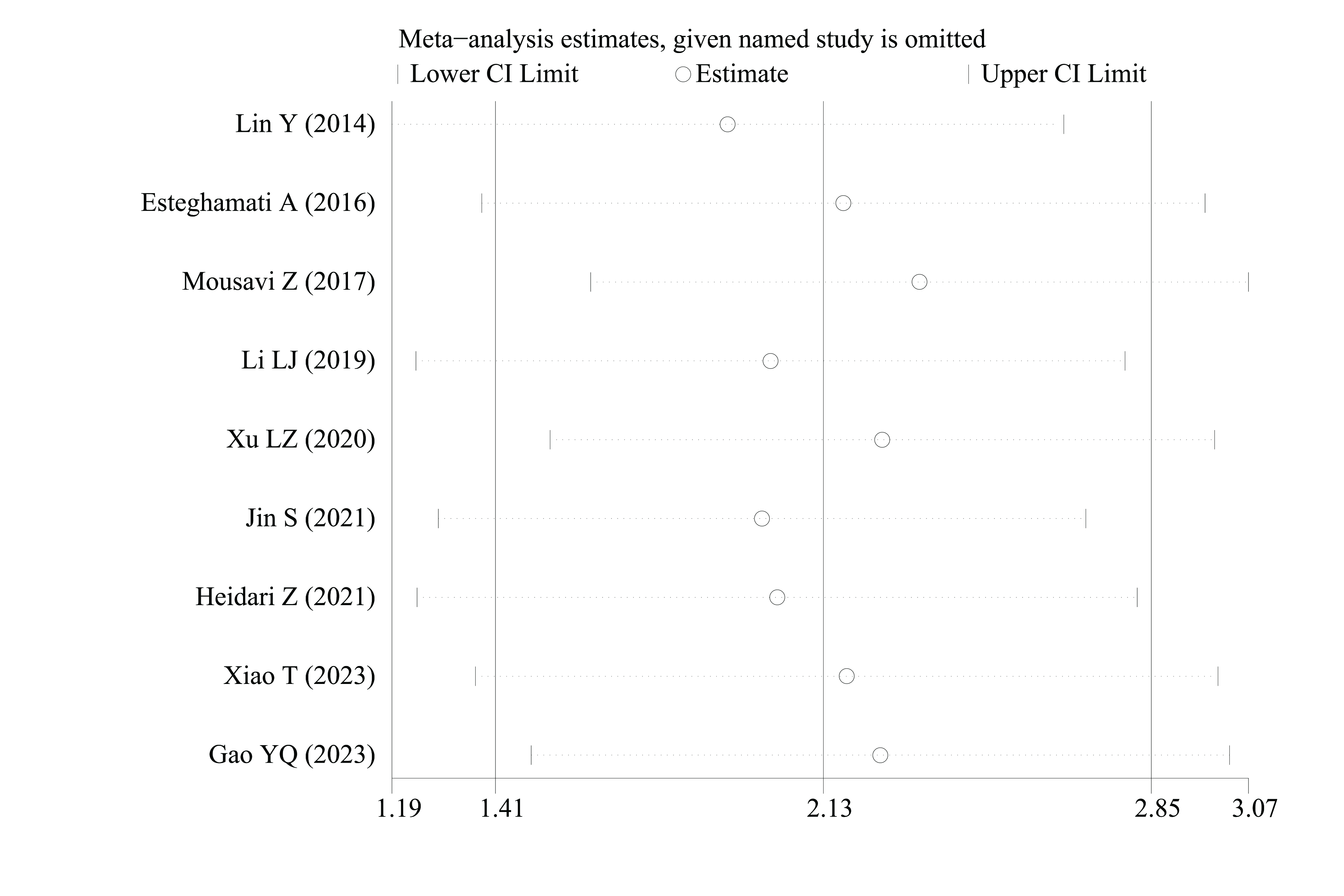

Supplement: Supplementary Figure 1 — The sensitivity analysis results of FGF21 level in patients with diabetic retinopathy compared to non-diabetic retinopathy diabetes patients. [file Image1.tif]

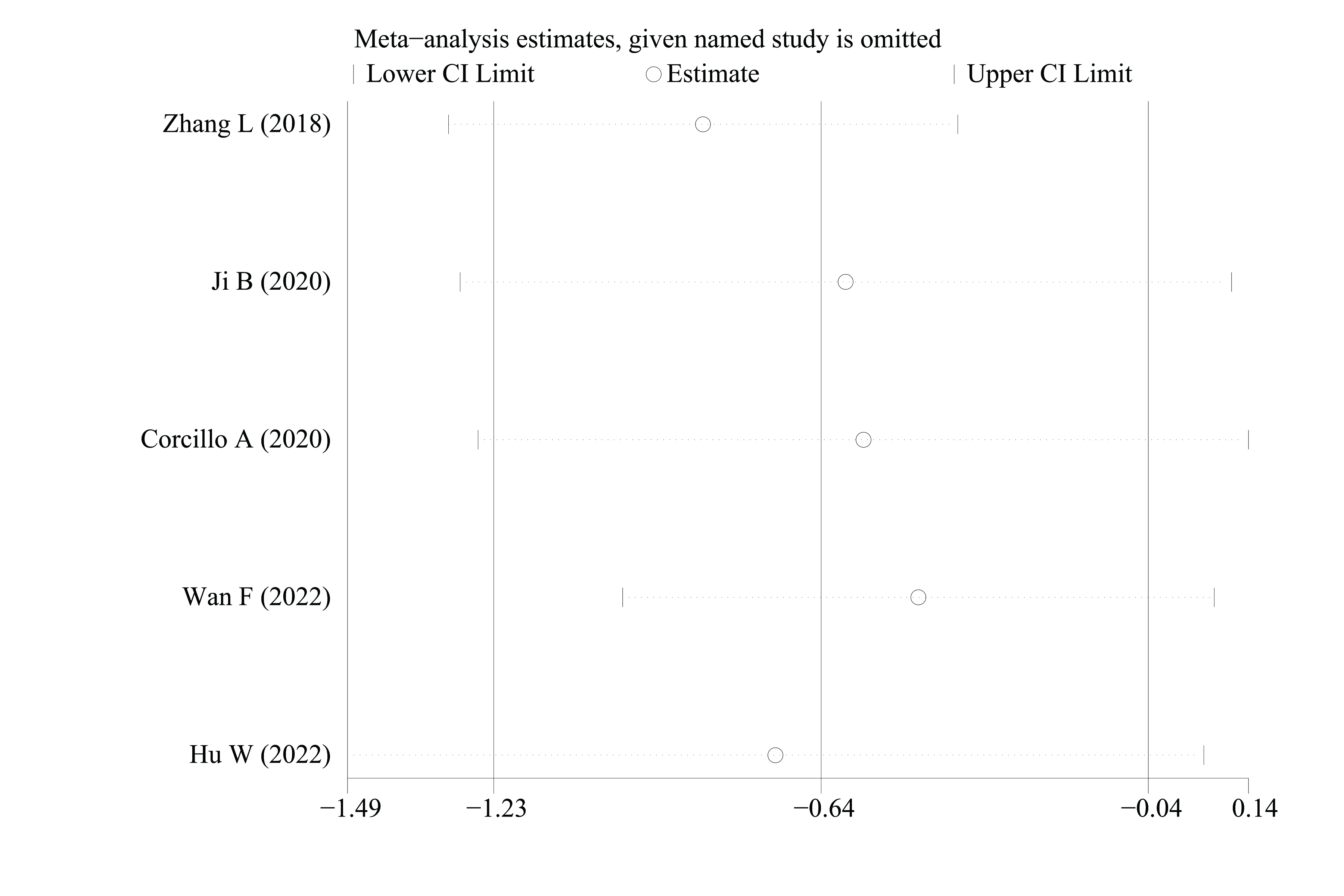

Supplement: Supplementary Figure 2 — The sensitivity analysis results of Klotho level in patients with diabetic retinopathy compared to non-diabetic retinopathy diabetes patients. [file Image2.tif]

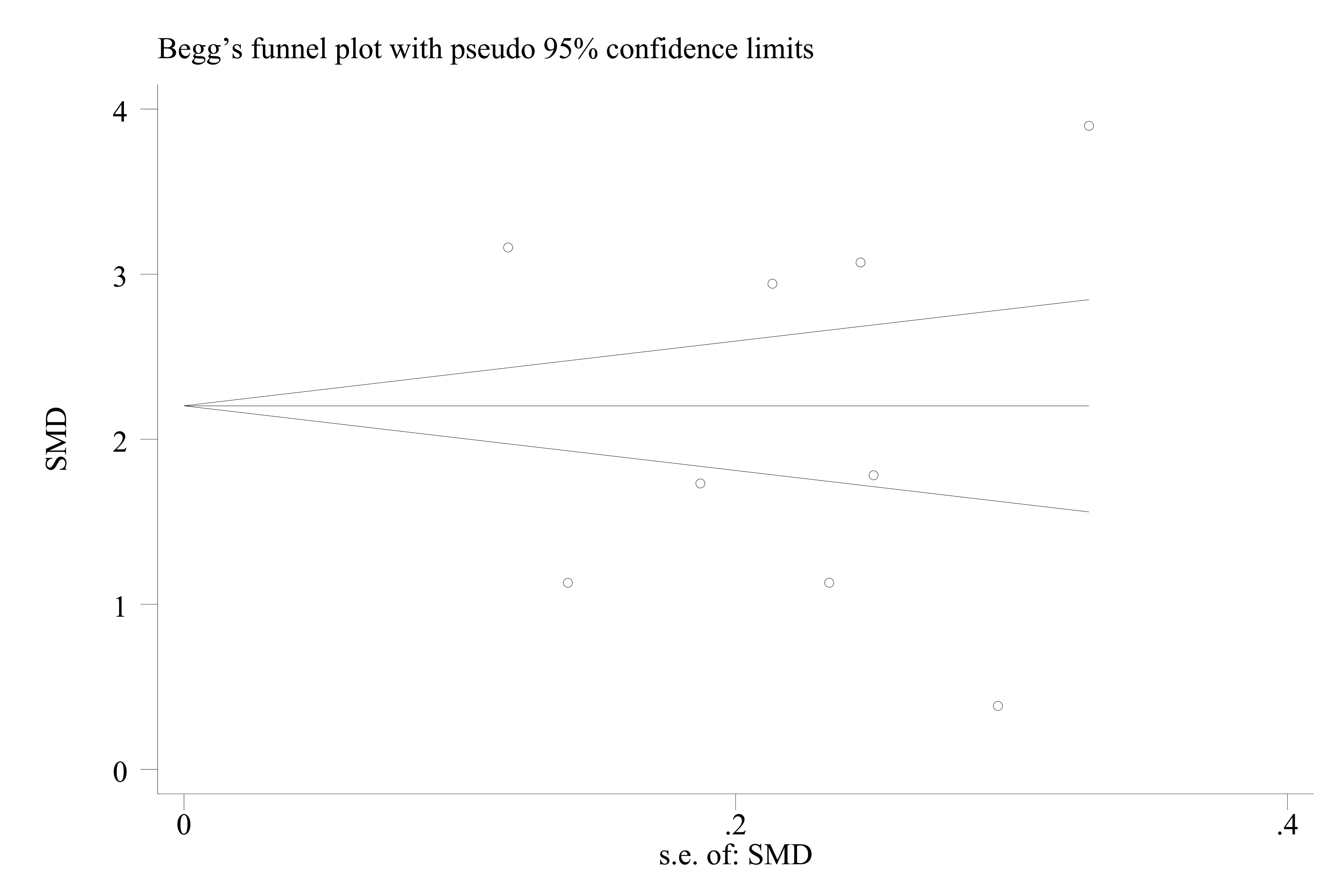

Supplement: Supplementary Figure 3 — The Begg’s test of FGF21 level in patients with diabetic retinopathy compared to non-diabetic retinopathy diabetes patients. [file Image3.tif]

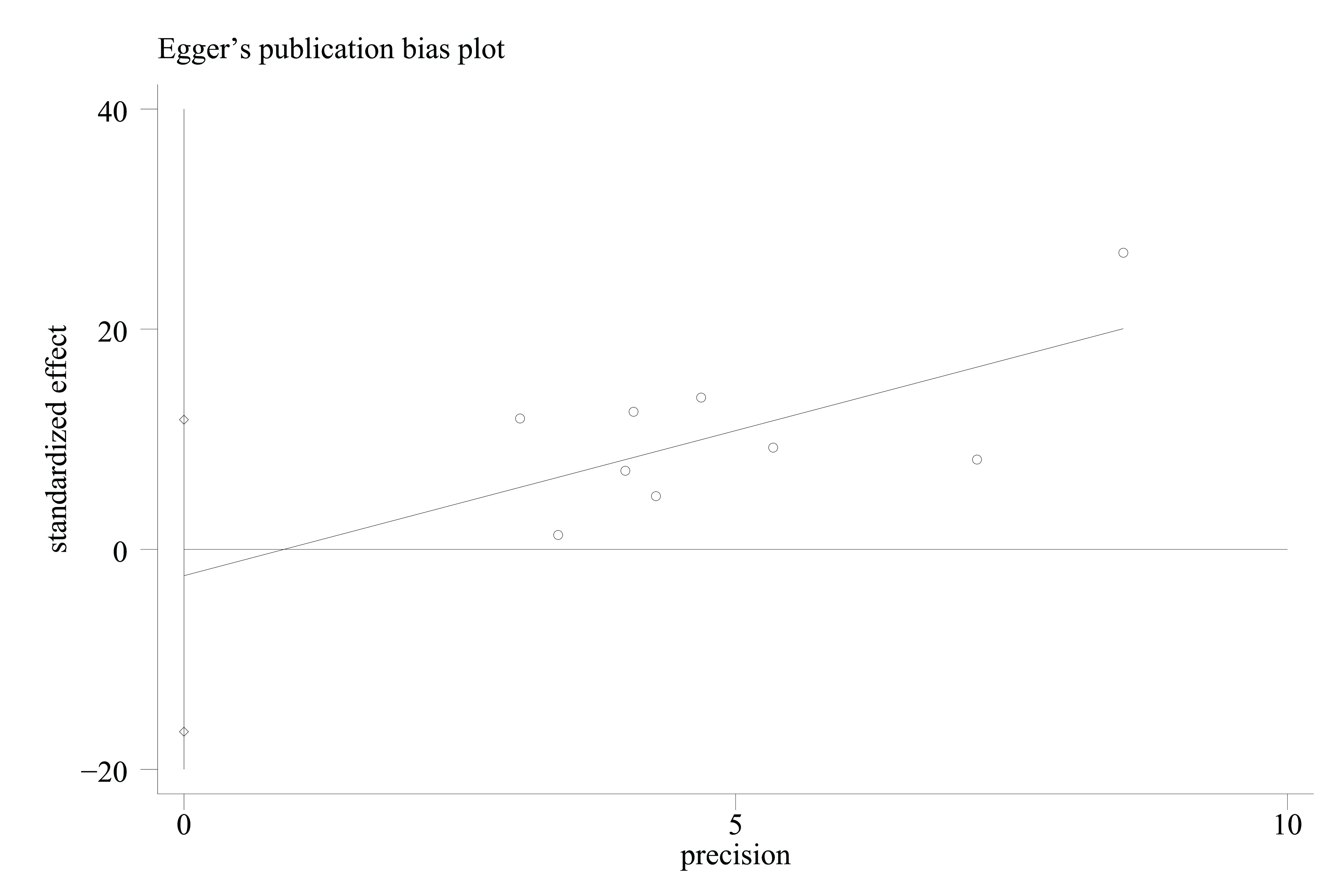

Supplement: Supplementary Figure 4 — The Egger’s test of FGF21 level in patients with diabetic retinopathy compared to non-diabetic retinopathy diabetes patients. [file Image4.tif]

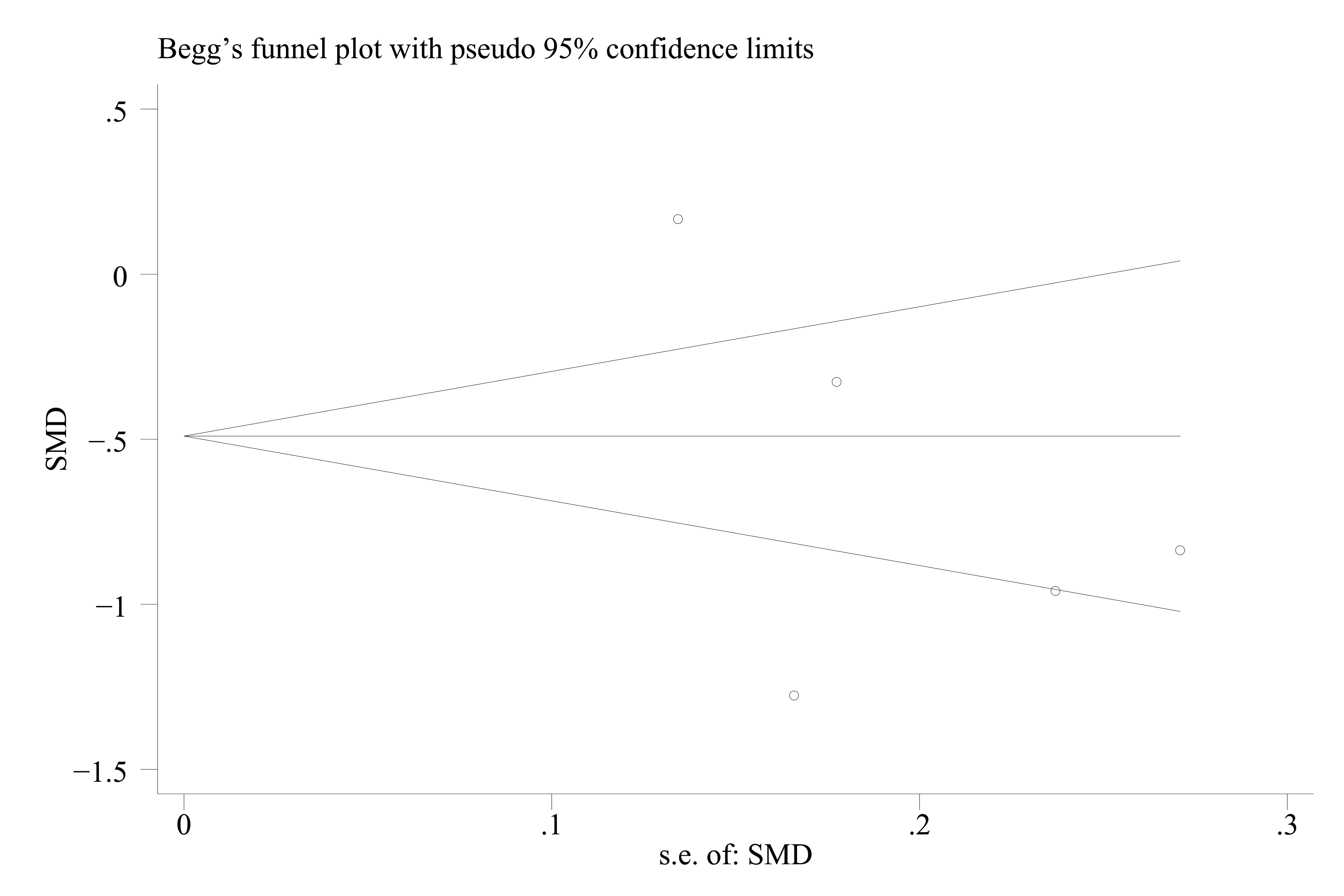

Supplement: Supplementary Figure 5 — The Begg’s test of Klotho level in patients with diabetic retinopathy compared to non-diabetic retinopathy diabetes patients. [file Image5.tif]

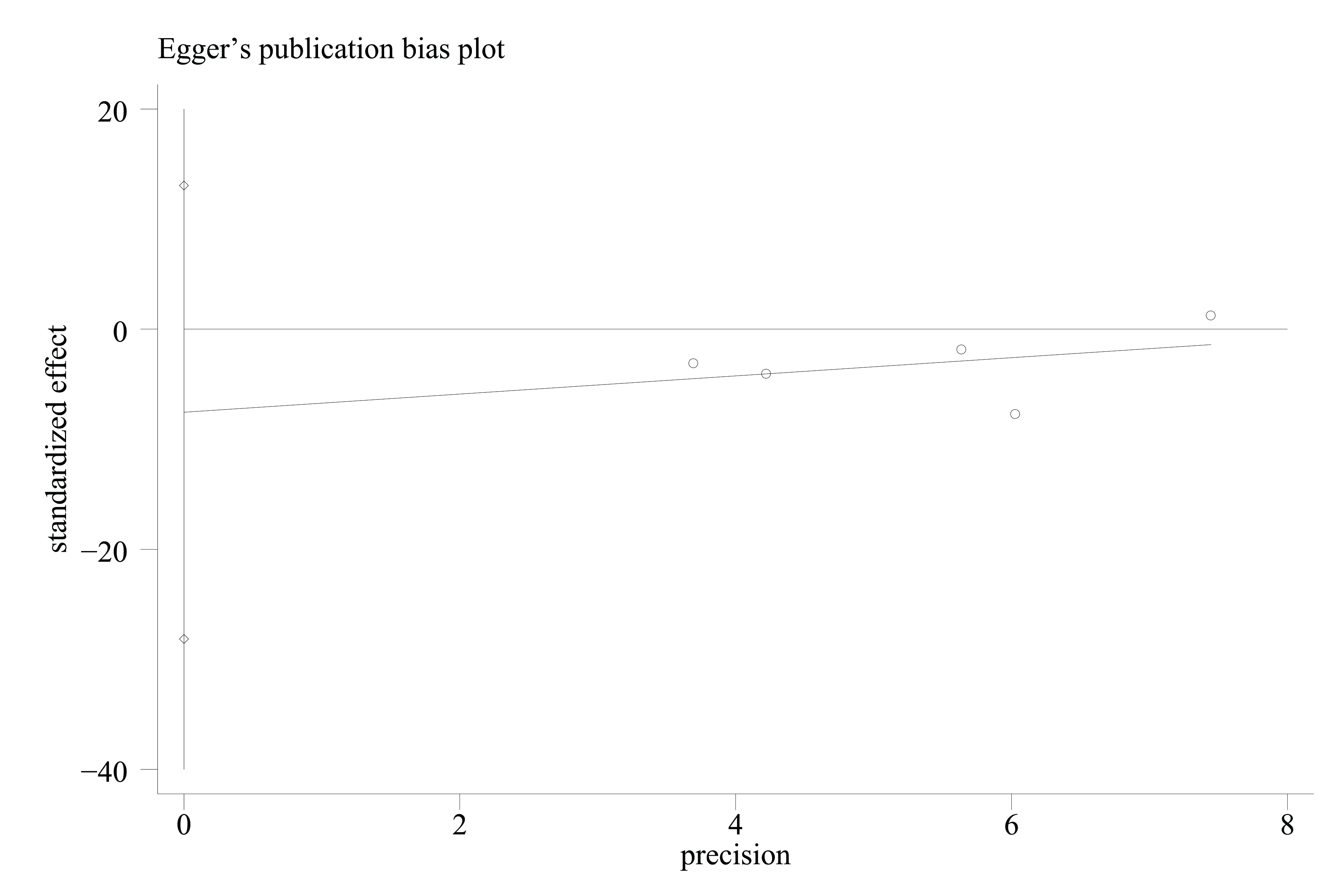

Supplement: Supplementary Figure 6 — The Egger’s test of Klotho level in patients with diabetic retinopathy compared to non-diabetic retinopathy diabetes patients. [file Image6.tif]
